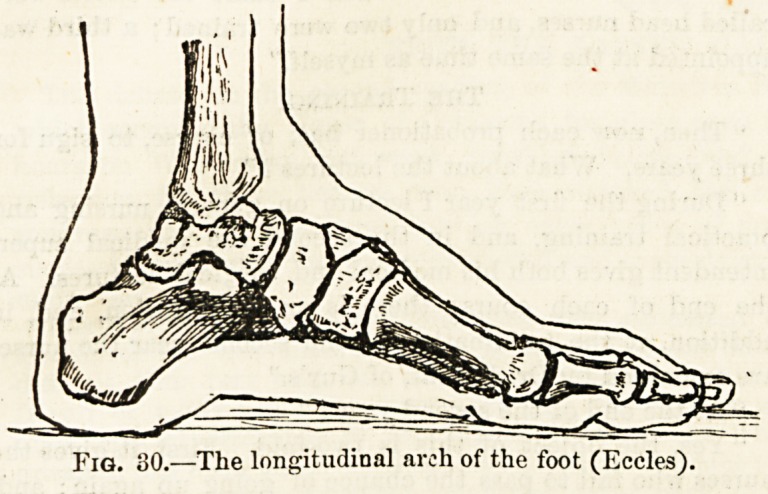# The Hospital. Nursing Section

**Published:** 1902-03-08

**Authors:** 


### The Hospital.

### Nursing Section.

Contributions for this Section of "The Hospital" should be addressed to the Editor, "The Hospital" Nursing Section, 28 & 29 Southampton Street, Strand, London, W.C.

No. 806.—Vol. XXXI. *SATURDAY, MARCH* 8, 1902.

### Notes on News from the Nursing World.

### ROYALTY AT KENSINGTON.

H.R.H. Princess Louise, Duchess of Argyll, President of the Kensington District Nursing Association, attended the annual meeting of that association on Tuesday afternoon, and the Duke of Argyll took the chair. In moving the adoption of the report the Duke observed that it recorded substantial progress in the past year, and was an earnest of much good work to be achieved in the near future. He mentioned that about £100 is yet required to finally clear the home at Bedford Gardens, and £300 is asked to furnish the new branch in Kenley Street. Sir Douglas Powell moved that the association deserved the heartiest support of the inhabitants of Kensington, and paid a well-merited tribute to the work of its nurses among the people whom they aided. After several other speeches had been delivered, the motion was adopted with enthusiasm.

### WORKHOUSE INFIRMARY NURSING ASSOCIATION.

An important meeting under the auspices of the Workhouse Infirmary Nursing Association will be held on Monday afternoon next, at 4 p.m., in Examination Hall, Victoria Embankment. Mr. William Bousfield will preside, and Miss Gibson, matron of the Birmingham Infirmary, will read a paper entitled "The Scarcity of Nurses in Country Workhouses; its Cause and Cure." Miss Gibson's own experience and position in the nursing world qualify her to speak with authority on the subject, and the general discussion which is invited should also prove to be illuminating. The twenty-second annual report of the Workhouse Infirmary Nursing Association, which has just been issued, does not contain any new information as to its work during the past year, of which our readers are ignorant; but the summary given of "some of the work done during the past twenty-one years," constitutes an interesting record. The total number of applications for nurses received by the Association was 1,391; the total number appointed was 844. The Association sent three deputations to the Local Government Board, and presented one memorial; two special interviews were held with members of the Local Government Board; and eighteen annual gatherings of nurses were held. It was not until the order on nursing was issued by the Local Government Board in August 1897, that the determination was arrived at to discontinue the work of training nurses, and since then the organisation has been indefatigable in urging the necessity of revising and strengthening the order. Its existence is maintained in the hope that it may be useful in furthering the questions which are still far from settled.

### THE YOUNGEST OF POOR LAW TRAINING SCHOOLS.

A pleasing picture of the nursing at a Poor Law Union Infirmary is given by our commissioner in another column, and the matron at Woolwich, which is the youngest of the workhouse infirmary training schools, must be congratulated upon a condition of affairs which so few in her position are able to achieve. There is one point which it seems advisable to emphasise. Nursing homes are not always needed for the sole purpose of making things more comfortable for the nursing staff. In the case of Woolwich, the number of beds for the patients was insufficient, and it became a matter of urgency to increase the supply. The erection of the new Home adds between twenty and thirty to the number. We rejoice, of course, in the better accommodation afforded to the nurses, who now, instead of sleeping three or four in a room, have each a room of their own. But it is due to the Woolwich Guardians to remember that the expenditure which the erection of the Home involved was partly prompted by a desire to provide more beds for sick paupers.

### THE WAR NURSES.

The *Avoca* has again arrived from South Africa, bringing the following nursing sisters as ship's staff: A. A. C. Higgs, who requires one month's leave and returns to South Africa; J. Stephenson; A. G. Rees; M. E. Buse; and M. Willington, who requires no leave and will return to South Africa. All these are members of the A.N.S.R. Miss H. G. Millar, A.N.S.R., was invalided home, and has been granted two months' sick leave. The *Orient* has just arrived from South Africa, bringing four nursing sisters, namely: E. Heaton-Cole, A.N.S.R., who requires one month's leave and returns to South Africa; B. Roberts, A.N.S.R., who is going to resign; Civil Nurses W. E. Cheesman, E. C. Lawrence, and M. A. Macready, all time-expired, and, as an invalid, Army Nursing Service Superintendent Nursing Sister S. E. Webb.

### WHAT IS AN UNSATISFACTORY REPORT?

An announcement was made by Dr. Ramsay, at the annual meeting of the York Home for Nurses, that the council felt justified in congratulating the supporters upon "the satisfactory character of the report." But as the funds of the home have in round numbers fallen off to the extent of £100 during the year, and the conduct of two nurses was reprehensible, one having to be dismissed, the congratulations seem a little out of place. It is true that the increase in the result of paid nursing work has been £169, which materially reduced the adverse balance of the general fund, but against this must be set the fact that the sick poor fund started in 1900 with £56 in hand, and finished the year with a deficit of £40. Again, though the health of the nurses has been good on the whole, one suffered severely from typhoid fever and was an invalid for three months, and another has been disabled since September owing to scarlet fever and complications. It is also admitted that the question of re-arranging and adding to the accommodation at the home has become urgent, and that there is a difficulty about finding the necessary money. We do not overlook the 1,184 cases nursed by the staff during the year ; but if the report of the York Home is considered satisfactory, it may reasonably be asked, What is an unsatisfactory one? At any rate the council seems to us to be thankful for small mercies, which is, perhaps, an admirable frame of mind.

### EAST LONDON NURSES AND SMALL-POX.

The annual meeting of the East London Nursing Society will take place on Tuesday next at the Mansion House. The funds are in an unusually prosperous state, owing to several unexpected receipts. Some of this balance can be invested, and a portion may be required to increase the number of the staff. During 1901 4,966 cases were undertaken, of which there were 3,462 recoveries, and probably many more amongst the 651 which had been removed to the hospital and elsewhere. Of the nurses themselves, one who had been assistant matron and matron is now working in the concentration camps, and another has been in the army hospitals in South Africa and has been awarded the distinction of the Royal Red Cross. With wise forethought all the nurses of the society were re-vaccinated in August last, so that they might be ready to render every possible help when the outbreak of small-pox came. They have been of much assistance in removing cases and doing all in their power to lessen the threatened evils of infection by advocating vaccination and sanitary precautions. No less than 23 of the London hospitals have shown their appreciation of the nurses' excellent work by sending notes of the treatment prescribed for their out-patients, which have been thus carried out till the next visit of the patients to the particular institutions at which they are treated.

### VOLUNTEERS AND NURSES.

Through the genial influence of the Medical Superintendent, Surgeon-Major Knott, and owing to the courtesy of the presidents of the Portsmouth Volunteer Corps, when giving their annual ball, many of the nursing staff of the Portsmouth Parish Infirmary have enjoyed an evening's good dancing. In an institution where such an amusement is necessarily restricted, the nurses all the more appreciated the kindness shown them in being so kindly remembered and so well looked after by the various M.C's.

### ACTION FOR BREACH OF PROMISE.

The case of McKenna *v.* Delany was settled at Dublin last week. It was an action for breach of promise of marriage, and the plaintiff was Miss Mary McKenna, a nurse at the Richmond Hospital, Dublin. The defendant was Mr. James Delany, county surveyor of King's County. When the case came before the King's Bench recently the defendant allowed judgment to go by default, and it was subsequently referred to Master Courtenay in order that damages might be assessed. A consultation having taken place between the counsel appearing for both parties, Mr. O'Shaughnessy, K.C., acting for Miss McKenna, announced that a settlement had been arrived at. "The terms were that Mr. Delany apologised for and withdrew any reflection made on the family of the plaintiff. The meaning of that was an observation made in an affidavit with reference to the plaintiff's family, and the defendant very properly withdrew and apologised. The defendant further stated that any derogatory observations with reference to plaintiff, if made, were made without his sanction, knowledge, or approval, and also that he never made any imputation on the honour or respectability of the plaintiff. The parties had consented to the assessment of damages at £500 and costs as between solicitor and client." We hope that this result is satisfactory to Miss McKenna.

### THE END OF THE BURLESQUE AT ARMAGH.

By degrees the Irish Boards of Guardians are realising that it is futile for them to continue to fight the Irish Local Government Board. Even the Armagh Guardians, having been advised by their own solicitor that they have not a leg to stand upon, have thrown up the sponge, and unanimously, but in a far from gracious manner, decided to appoint an assistant nurse and two wardmaids in accordance with the special order of the Local Government Board, dated December 4th, 1901. The chairman, in proposing that the Guardians should submit, declared that they had fought the question in the interests of the ratepayers, and to save the public the expense of the services of costly and unnecessary officials, whereupon Mr. Lavery cruelly retorted that the legal costs incurred had no justification, "as the Local Government Board had the power of the law behind them." He thought that it was time "to end the burlesque." But the mistake was to begin it.

### MISS FORSYTHE'S ACTION.

The action brought by Miss Charlotte Forsythe, a nurse, who in 1888 held a position at Queen Charlotte's Lying-in Hospital, against Dr. Law, her former medical attendant, occupied Mr. Justice Grantham and the Court for several days last week, and was then stopped by the jury, and a verdict given for the defendant. Miss Forsythe alleged that Dr. Law administered morphia, chloral, cocaine, and other drugs to her in such quantities as to cause her to lose her reason and injure her health. Dr. Law had employed Miss Forsythe about 1888, but afterwards he was introduced to her family and became on friendly terms with them. She had been more or less under his treatment for asthma and other ailments till quite recently. Miss Eliza Critchlow, described as a trained nurse, gave evidence that she nursed Miss Forsythe at Bath ten years ago, and saw her again in 1899. She stated that she felt very strongly about the use of morphia, and believed the plaintiff at that time to be dying of drugs "and things given her." We allude to the case because the plaintiff is or was a nurse ; but the question at issue in no way affects the relation between doctors and nurses in their professional capacity. The simple legal point raised was whether the treatment given by a medical man to his patient was right or wrong.

### FEMALE NURSES FOR MALE LUNATICS.

It is interesting to note the result of the experiment started five years ago at the Long Island, New York, State Hospital, of employing women nurses in the male wards. A marked improvement has been observed in the general order. The majority of insane men seem to respect a woman, and manifest a sense of deference towards her which is not usual in their treatment of male nurses. The fact of their presence has also, it is stated, been a source of satisfaction and comfort to women relatives visiting the men patients, and naturally such housekeeping duties as fall to the nurses' lot are much better performed under the auspices of the women than under those of the men. Seven out of the ten wards now have a woman attached to them.

### A HANDSOME PROFIT AT BRISTOL.

In congratulating the committee of the Bristol Nurses' Institution upon the "very handsome profit" on the year's working, Dr. Shingleton Smith was well within the mark. The difference between the receipts and expenditure amounted to no less than £615, of which £281 was made by the institute, and £334 by the nursing home. It is satisfactory to learn that in these gratifying circumstances the committee decided to pay to the indoor nursing staff, in addition to the usual bonus, sums varying from £2, according to the length of their services to the home. This absorbs about £150 of the profit, and still leaves a substantial sum to the good. The number of nurses employed by the institution is now 42, and during the last twelve months 770 cases were nursed. Altogether, the position supplies an object lesson of the possibilities open to such an organisation in a popular centre.

### MISS STONE'S COMPANION.

An interesting point in connection with the capture and release of Miss Stone is that her companion, Madame Zilka, is described as having been a hospital nurse before her marriage. Madame Zilka was more to be pitied even than Miss Stone because of her condition. The little girl was born after a ten hours' ride on horseback, the mother smothering its cries so that the brigands might not know what was happening. She had no comforts, no attendance, nor anything needed at such a time, only a screen and a rough shelter to protect her from the cold. Three days after the birth, the mother, the baby, and Miss Stone were again on the move, and it is stated that but for the knowledge which Madame Zilka had acquired during her hospital training, both she and the child would probably have lost their lives. The baby's garments were made from odds and ends of clothing begged from the brigands.

### A NURSE'S ESTATE.

Miss Conway, formerly sister of "Whitbread Ward" at the Middlesex, who has recently died, was, after her retirement from active work, a pensioner of the hospital. It is gratifying, says the annual report of the weekly board, to note that Miss Conway has bequeathed the residue of her estate, amounting to £131 13s. 2d., for the benefit of the cancer wards, where she had laboured for over twenty years.

### PRAISE FOR BELFAST INFIRMARY NURSES.

The Society for Providing Nurses for the Sick Poor at Belfast, which has just held its annual meeting, finding that the staff of 10 nurses was not fully employed, distributed circulars in June amongst the doctors calling their attention to the organisation. As the result there was an increase of 120 cases over the number reported in the corresponding six months of the previous year. Of the 139 typhoid cases which were nursed only 16 died, whereas of the consumptive patients only 55 were still alive out of the 198 nursed during the year. Allusion was made in the report to the depressing effect of nursing these helpless cases. There had been two vacancies during the year, one nurse leaving to be married, the other for private work. None but nurses with three years' training are admitted to the staff, many of them coming from the Belfast Union Infirmary. These, it was stated, were usually found most efficient and suitable in character and training. The balance in the bank at the end of 1901 was quite satisfactory. Two nurses still continue to receive their pensions from the superannuation fund, and £23 was paid in to the Royal National Pension Fund during the year.

### HEALTH MISSIONERS.

At a meeting on Monday in support of the Women's Memorial to Queen Victoria, held by invitation at 29 Park Crescent, W., the chair was taken by the Lady Mayoress, who said that the Queen's Nurses were not only the means of saving lives, but they also taught the poor habits of cleanliness in their own homes. Mr. Ernest Flower, M.P., one of the honorary secretaries of the Memorial Fund, dwelt on the educational value of the movement. "Miss Florence Nightingale had spoken once of the nurses as 'health missioners,' and he did not think that a better name than that could be found for them." Dr. Adler, the Chief Rabbi, spoke on behalf of the fund, and Mr. Fleetwood H. Williams, the assistant secretary of the fund gave some interesting details as to the number of nurses that were required.

### AN INTERESTING COMPARISON.

Four years ago the Abergele and Pensarn District Nursing Association was formed, and we have before us copies of the report issued at the end of the first twelve months and of the report for 1901. In 1898 the number of patients received was 47, and the number of visits paid 2,226. Last year the number of cases was 86, and the number of visits 3,420. The receipts of the association have also increased, and the committee are so satisfied with the work done by the nurse, that they have decided to raise her salary. Considering that there are only about 2,000 inhabitants in the town of Abergele, it may fairly be said that the support given to the association is highly creditable.

### SHORT ITEMS.

The recent exhibition of British Silks and Coronation Robes at the Hanover Gallery, by Messrs. Liberty and Co., realised £74 12s., which has been sent to the manager of the fund for the Women's Memorial to Queen Victoria.—Last Thursday evening an enjoyable entertainment was given to the in-patients of the Cancer Hospital at Brompton, the programme being arranged by Miss C. E. Brownfield.—The next examination of the Medico-Psychological Association will be held on May 5th. Schedules which must be filled up by all intending candidates and returned before April 7th, can be obtained from Dr. Miller, Hatton Asylum, near Warwick.—The first three-yearly examination of St. Leonard's Infirmary, Shoreditch, was held on Tuesday and Friday last week, the examiner being Mr. Stephen Paget. Eleven nurses entered and all passed very creditably. The papers were practical and well chosen.

### Lectures to Nurses on Anatomy.

By W. Johnson Smith, F.R.C.S., Principal Medical Officer, Seamen's Hospital, Greenwich.

### LECTURE XII.—BONES OF THE LOWER LIMB.

In examining the thigh-bone or *femur* (figs. 1, [10]), the largest and longest bone in the human skeleton, the following points should be carefully noted:—On looking at the bone in its place in a complete skeleton it will be seen that it does not hang straight down and parallel to the middle line of the body, like the humerus, but that its direction is oblique, the upper ends of the two bones being widely separated by the pelvis, whilst the lower ends almost touch

Fig. 28.—Upper extremity of right thigh-bone showing the head, neck, and two trochanters.

one another at the knee. In consequence of the greater width of the female pelvis this obliquity is more marked in women than in men.

The long, rounded shaft of the femur differs from the straight shaft of the humerus in being curved forwards and a little outwards. This curvature is much exaggerated in rickety subjects.

The smooth and globular head (fig. 28), the contour of which forms nearly two-thirds of a regular circle, fits closely and securely into the acetabulum of the corresponding innominate bone, which is deepened by a horse-shoe shaped rim of gristle called the *cotyloid* cartilage. The articular cartilage covering the head of the bone is deeply pitted near its centre. This depression corresponds to the insertion into the bony structure of the head of a flattened band of fibrous tissue—the *ligamentum teres*.

The neck of the femur, like the head, really merits the name. It is a thick and strong rounded process of bone, at least an inch in length, which takes a more or less slanting direction in its relation to the shaft of the bone. This slanting direction is less marked in the female than in the male, and also diminishes as age advances, the neck being more in a line with the shaft in the infant, less oblique in the adult, and set almost at right angles in the aged.

The neck is set on two shoulders called *trochanters*, which serve for the attachments of the muscles that turn the lower limb outwards or inwards—evert or invert. The outer, forming a large square process which can be readily felt under the skin and is the most prominent part of the hip, is called the *great trochanter* (fig. 28 A) in contradistinction to a much smaller process on the inner side, called the lesser trochanter (B).

The lower end of the femur is expanded into a broad and thick articular extremity divided by a shallow notch in front, and a deep gap behind, into the two condyles. If the shaft of the bone be held quite upright it will be seen that the condyle on the inside projects downwards more than the external condyle. This irregularity enables the whole width of the lower end of the bone to be brought in contact with the level surface at the upper end of the large bone of the leg, and thus serves to compensate for the oblique direction of the thigh bone.

In the leg, as in the fore-arm, there are two long bones, one thick, strong, and massive, on the inner side, the *tibia* (figs. 1, [12]); and a slender and apparently useless and rudimentary bone, the *fibula* ([13]), on the outer side. From the lower end of each of these bones projects a distinct bony process, that from the fibula being the larger and more prominent. That attached to the tibia is called the *internal*, and that attached to the fibula the *external malleolus*. These two processes form the lateral boundaries of the ankle-joint, and confine the foot so that this extremity can only be flexed or extended, and cannot be turned inwards or outwards. The expanded upper end or head of the tibia alone forms the lower part of the knee-joint, the fibula taking no part. Below, at the ankle, the tibia takes a much larger share than the fibula in the formation of this joint. The former bone, therefore, may be regarded as the main and almost exclusive support of the weight of the body afforded by the leg.

A very common injury of the leg is fracture of the fibula near the ankle, with fracture of just the tip of the internal malleolus or rupture of the ligament extending from this process to the foot. This injury which is marked by a characteristic twist of the foot is termed *Pott's fracture*.

The bones of the foot called the tarsal bones, which correspond in position to the carpal bones of the hand, are much larger than these, and are seven—not eight—in number. Moreover they are arranged irregularly, and not in two rows. Behind there is one large bone forming the heel, which is

Fig. 29.—Skeleton of right foot.

termed the *calcaneum* or *heel-bone* (fig. 29, [1]). Surmounting this is the *astragulus* (2), the only bone of the group which takes part in the ankle-joint. The *calcaneum* articulates in front with a bone which, on account of its cubical figure, is called the *cuboid bone* (4); and the astragalus with a bone called, from its supposed resemblance to a boat, the *scaphoid* bone (3). The outer of these bones—the cuboid—articulates with two of the long bones of the toes, but between the scaphoid and the three inner long bones are interposed three other tarsal bones each of a wedge-like shape, and for this reason called the cuneiform bones (5).

In front of these seven tarsal bones are five long bones, all longer and more massive than the bones of the palm of the hand, termed *metatarsal* bones. Completing the skeleton of the foot we find here, as in the upper extremity, fourteen phalanges, two for the great toe and three for each of the other toes.

The bones of the foot with their numerous joints and buffers of yielding cartilage and their ligaments form two yielding and springy arches—one the longitudinal arch (fig. 30), most marked on the inner side, supported behind by the heel-bone and in front by the ball of the great toe, and an arch from side to side corresponding to the regular curve across the top of the foot and to the transverse hollow of the sole. Thus when the weight of the body is thrown on the lower extremity in standing, or in locomoture, the foot becomes longer and broader. The foot, in a long struggle with those

FIG. 30.—The longitudinal arch of the foot (Eccles).

conditions of modern civilisation which tend so much to restrict and abolish its movements, has, in comparison with the broad foot of ancient sculptors, become a stunted and crippled limb.

So far we find that as there are eight carpal and seven tarsal bones, there is one bone less in the lower than in the upper limb. This numerical discrepancy, however, is made up by the presence in front of the knee-joint of a flat triangular bone well known as the patella or kneecap pan (figs. 1, 11), which, though it resembles in some respects the olecranon process of the ulna, has no corresponding loose bone in the arm.

In front of the joint between the metacarpal bone and the first phalanx of the thumb, and also on the under surface of the corresponding region of the great toe are two small rounded and disc-shaped bones, which are closely attached to the tendons of the muscles bending or flexing the thumb and toe. These are called *sesamoid* bones.

### Wants and Workers.

THE HOSPITAL.—Nurse Deane, Nurses' Home, Cobham, Surrey, will be pleased to forward THE HOSPITAL, on receipt of postage, to any nurse the Monday after publication.

### To Nurses.

**WE invite contributions from any of our readers, and shall be glad to pay for "Notes on News from the Nursing World," or for articles describing nursing experiences, or dealing with any nursing question from an original point of view. The minimum payment for contributions is 5s., but we welcome interesting contributions of a column, or a page, in length. It may be added that notices of appointments, entertainments, presentations, and deaths are not paid for, but that we are always glad to receive them. All rejected manuscripts are returned in due course, and all payments for manuscripts used are made as early as possible after the beginning of each quarter.**

### "A Word of Welcome."

THE following verses were addressed to H.R.H. Princess Christian of Schleswig-Holstein, on the occasion of her visiting the Royal Free Hospital, and presiding at the annual Court of Governors last week:—

Welcome! we bid you welcome, gracious Princess, here to-day,
And a few brief words of greeting, with your leave, we fain would say;
Words of very simple rhyming, phrased with no poetic art
Yet, haply to be listened to, as coming from the heart.

Your presence is most precious, for it fails not to recall
The name of the Good Queen, who lived so dearly loved by all;
And by none with greater reason than the poor folk you may see
At Hospitals like this, which She well named the "ROYAL FREE."

The poor sick folk! ah, Princess! 'tis their need now brings you here,
'Tis your sympathy in suffering—the moving cause is clear;
True sympathy with sufferers! 'tis the holiest power we know,
For it came from Christ Himself, to help His creatures here below.

Who founded all our Hospitals, our Homes for sick and poor?
Who, but He, the Great Physician, who went forth from door to door,
Ever helpful to the helpless, ever comforting the sad,
Healing sick, and blind, and crippled, turning grieved hearts into glad.

Men now feebly strive to follow Him; fair Science brings her aid;
Great onward strides in Science through our Hospitals are made;
In the noble work of nursing, too, what progress have we seen
Since the day when Florence Nightingale first bowed to her dear Queen!

The noble work of nursing! What in man's work can compare
With the patience, skill, self-sacrifice, and bravery shown there?
Shown alike with equal kindness, equal care and wisdom, shown
To the pauper in the hovel, or the King upon the throne.

And who now with more fitness to our Hospital should come,
Than the gentle Princess-President of the British Nurses' Home?
The nurses who by hundreds have so bravely gone afar,
To serve and save our soldiers, in the ghastly land of war.

What great folk do the lesser folk will prattle of, 'tis said,
And good work well may prosper, when the news of it is spread:
So, golden streams, Pactolus-like, fresh flowing we may see
From the day when the good Princess came, to view the "ROYAL FREE."

H. S.

### Death in Our Ranks.

ST. BARTHOLOMEW'S HOSPITAL.—We regret to learn of the death on Friday last week from pleurisy, after only four days' illness, of Miss Helen Margaret Buckland, staff nurse at St. Bartholomew's Hospital. Miss Buckland was 34.

### The Nurses of Woolwich Union Infirmary.

A CHAT WITH THE MATRON (BY OUR COMMISSIONER).

The selection of Woolwich Union Infirmary for a visit last week was determined by the fact that a new nursing home has just been opened at Plumstead, and also by a desire to ascertain the result of the foundation of the training school which was started under the auspices of Miss Helena Gooding, the present matron, less than three years ago. On my arrival Miss Gooding showed me over the home, which stands on very high ground on a level with Plumstead Common, and commands a remarkably varied view of water, woods, and chimneys. It is a distinct ascent from the infirmary to the new building, though the distance is short. The nurses are only too glad to have their home, which is certainly of the best, and reflects great credit on the Woolwich Guardians, the architect, and all concerned.

### The Accommodation.

"How many rooms does it contain?" I inquired, as we inspected an exceedingly comfortable room on the first floor.

"There is a bedroom for every nurse, and there are four sitting-rooms—one for the assistant matron, one for the night sister, one for the ward sisters, one for the probationers, and a general room for the use of the whole staff, for dancing or other purposes of recreation, including ping-pong."

We went into each of the sitting-rooms, which are all of a fair size, suitably but not extravagantly furnished. The general room, which contains two fireplaces, is very commodious. I noticed a handsome piano in the corner, and asked if it was a recent importation.

"No," replied the matron, "it came from the old buiding, and was the gift of some of the guardians in their private capacity. As you see, the tennis lawn has yet to be made. But the guardians hope to provide both a tennis and a croquet lawn."

"Meanwhile," I rejoined, "you have a charming balcony."

"Yes, we consider that a pleasing feature. Also you will have noticed that the staircases and corridors are warmed by hot water pipes, that there is ample bath-room accommodation, and that the night nurses have the top corridor to themselves."

"That is a most necessary arrangement, and I observe that the corridor is shut off by a swing door. But where do the nurses dine?"

"In the infirmary. No meals whatever are served in the home. I think that this is a great advantage. It enables the nurses to have the building entirely to themselves."

"Is the building fire-proof?"

"Entirely. The floors are all concrete, with blocks laid on them. The home, which was commenced a little over 18 months since, has been a recognised want for a great number of years."

### The Old Order of Things.

"What were the sleeping arrangements before the home was built?" I asked, as we returned to the matron's sitting-room in the infirmary.

"The nurses were sleeping three, and even four, in a room. They were comfortable enough, but there was no privacy, and it was awkward when girls who did not like one another had to share rooms. But the idea of the Guardians in building the home was not only to afford better accommodation for the nurses, it was also to give extra space to the patients."

"How many more patients shall you be able to receive as the result of the improvement?"

"Between twenty and thirty. Hitherto the number of beds has been 280, it will now exceed 300."

### The Beginning of the School.

"How soon after you came here was it decided to make the infirmary a training school?"

"I began to give lectures six weeks after I took up my duties as matron. The proposal was made by the Guardians, and the Local Government Board immediately sanctioned it. The former matron, who resigned on account of failing health, was much liked, but the Guardians thought that with the necessary change a different system should be introduced."

"You came from the London Hospital?"

"Yes, I was trained at the London, and was afterwards sister there for three years. Our first certificates will be issued in November."

"How many probationers did you find here?"

"Twenty. There are now thirty-two in different periods of their three years' training, six ward sisters, a night sister, and an assistant matron. When I came the sisters were called head nurses, and only two were trained; a third was appointed at the same time as myself."

### The Training.

"Then, now each probationer has, of course, to sign for three years. What about the lectures?"

"During the first year I lecture on general nursing and practical training, and in the second the medical superintendent gives both his medical and surgical lectures. At the end of each course there is an examination, and, in addition, at the termination of the second year the nurses are examined by Dr. Bryant, of Guy's."

"At the end of the second year?"

"Yes, the object of this is two-fold. First, it gives the nurses who fail to pass the chance of going up again; and, on the other hand, it enables those who pass to devote their undivided attention to work in the wards. The final examination is written and oral. Dr. Bryant sends the written questions, and the answers to these being corrected by him, he comes the day following for the oral examination. We were very fortunate at the last examination held by Dr. Bryant. The next is in June."

"Do you experience difficulties in obtaining nurses?"

"I have only had to advertise once lately, and then there were more than fifty applications, from amongst which I was able to get the twelve I wanted."

### Hours and Holidays.

"Have you made any changes in the off duty time?"

"I was able to increase the off duty time of the sisters when the home was opened. They now have half a day off, from 2 to 10 p.m. every week for three weeks, and then from 7 a.m. to 10 p.m. the fourth week. They are allowed to sleep at home the night before their day off duty."

"How long are they on duty?"

"They come on at 8 a.m. and go off at 9 p.m. As well as meal times, they have two hours off duty during the day They have three weeks holiday in the year."

"And the probationers?"

"The probationers have half a day off at the end of one fortnight and a day the other fortnight. They are on duty from 7 a.m. until 8 p.m., and they get the same time off duty during the day as the sisters, with a fortnight's holiday."

"What salaries do you pay them?"

"For the first year £12; for the second £15; and for the third £18. They are always paid when they are ill, but they have to make up the time for training."

### The Night Nurses.

"How many nurses are on duty at night?"

"Ten have just been sanctioned by the Local Government Board. We have been having eight. There are two wards in each block, and three nurses are allotted to each block, with one to spare. I do not like the wards left for five minutes at night, and with the present staff there is no reason why they should be left. The night nurses come on duty at 8 P.M. and go off at 7.30 A.M. They have a night off once a month, and they also go out from 8 to 11 every morning. Their work is not, I consider, so hard as that of the day nurses."

"Have you had any trouble about their food?"

"None at all. They have the same diet as the day nurses. Speaking of the food generally, we can always have what we require."

"Do you issue Sunday passes?"

"The nurses have them alternate Sundays, from six to ten. There are services in the chapel every Sunday, and once a month there is a celebration of the Holy Communion at eight. But the passes afford them the opportunity of attending any of the churches.

"The guardians provide indoor uniform?"

"Yes, but not outdoor, the wearing of which is optional. Most of the nurses, however, provide outdoor uniform for themselves. They find it convenient and economical. It is optional for the nurses to contribute to the superannuation fund, and most of them contract out of it. When they have completed their training, they do not always remain in the Poor Law, in which case the money deducted from their salaries would be lost.

### The Midwives Bill.

FROM THE LADIES' GALLERY (BY A CORRESPONDENT).

THE debate on the second reading of the Midwives Bill, which occupied the House of Commons for close upon five hours on Wednesday last week, provided ample food for reflection for those hearers who have watched the rise, progress, and final collapse into an early grave of many successive measures for the registration and supervision of midwives.

Introduced by Lord Cecil Manners, who addressed the House for the first time, and who accomplished his rather difficult task very well, Mr. de Tatton Egerton, a faithful friend of many years' standing, seconded the motion, and drew special attention to the length of time the whole question has been under consideration, and to the fact that legislation in other European countries dates back to the early years of the last century.

It was impossible not to feel that every word uttered by Sir John Batty Tuke, who opposed the Bill as lacking in strength and purpose, and, by the omission of the "penal clause" preventing uncertificated practice as well as the use of the title of midwife, as "futile, and to a certain extent mischievous," was strengthening the arguments of those who wished to see even the present Bill passed, rather than none. His speech was curiously ineffective from the point of view of the opposition, and did not move the House in the least.

A very unnecessarily unpleasant speech, quite beside the question, was made by Sir Barrington Simeon, whose argument that the control and supervision of midwives would lead to an increase in the practice of illegal operations must have been inspired by some singularly illogical mind.

Dr. Farquharson ranged himself altogether on the side of the Bill, contending that it was in the direction of making the midwife of the future clean and antiseptic, and "though not perfect . . . a serious and honest attempt to regulate, and perhaps remove, a great and crushing evil."

Mr. Griffith Boscawen also supported the second reading, while urging the restoration of the penal clause, and Mr. Wason earned the gratitude of every woman in the country by his admission that "this was essentially a woman's question," and as women were unrepresented in Parliament he thought it was incumbent on members to do all they could to protect their interests.

Other supporters of the Bill were Mr. W. Lawrence, who said he had been pressed by important bodies in Liverpool to advance the Bill in any way he could, Mr. Emmott, Mr. Luke White, who spoke strongly in favour from the coroners' point of view, Sir Savile Crossley, Mr. Burns, Sir Michael Foster, the Home Secretary, and, of course, Mr. Heywood Johnstone, whose "ewe lamb" the Bill is acknowledged to be in a special manner.

Undoubtedly, the most effective speech on behalf of allowing the Bill to pass the second reading and be referred to a committee was made by Sir Michael Foster, whose temperate, well-reasoned, and humane remarks clearly carried the weight they deserved. A medical man, and the son of a medical man, Sir Michael spoke with an authority which could hardly be gainsaid, and there was no doubt that the House was with him in his finely-put allusion to the "travail" of childbirth, a journey fraught with the utmost peril to life. The Bill, he said, was in the interest of the poor in order that they might carry with them on that journey not a doctor, but one who knew when the doctor should be sent for. "The chief qualifications for a midwife were three—first, to know when to send for the doctor; second, to be cleanly, and to know what cleanliness means; and third, never to drink."

A clever but distinctly unconvincing speech was delivered by Sir Walter Foster, who made the most of the objections that may very fitly from various points of view be urged against the present measure, especially taking the ground that it would open the door to "unqualified practice," and cause injustice to medical students, while giving a status to women taking out licences as midwives. He urged that the Bill should be referred to a Select Committee, and this suggestion was seconded by Sir John Tuke, but was negatived, and the Bill was duly read a second time. Finally, on the motion of Mr. Heywood Johnstone, it was referred to the Standing Committee on Law. Other speakers against the Bill were Dr. Ambrose and Mr. T P. O'Connor.

Behind the grille of the Ladies' Gallery the debate was listened to with interest, not untempered with mirth, at the deliciously naïve ignorance of this "woman's question" occasionally exhibited by the legislators below. It was amusing to hear the "Royal Nurses' Association" quoted as a great and important body entitled to representation on the Central Midwives' Board, and one wondered what it might be—the Queen Victoria Jubilee Institute, perhaps, a body which might with some reason claim such a right, seeing the number of district midwives amongst its ranks. Mr. T. P. O'Connor, who filled out time in a manner which was very clever, had clearly been primed from the same source as some of the other members, and the truth was out when a letter from the Hon. Secretary of the Royal British Nurses' Association was produced, though it was hardly apparent why an association which has nothing to do with midwives should desire such representation. The familiar old bogey of an insufficient "two-month training" was trotted out by Dr. Ambrose, an Irish member; but Mr. Johnstone, ever watchful, got home promptly by a demand as to where in the Bill this period of time was mentioned. Considering the number of members in the House who knew well the important part the Midwives' Institute has played in the demand for legislation these many years past, it was more than a little odd to hear opposing members insisting upon the non-existence of that incorporated body to which trained midwives owe so much; and there was a fine "confusion of epitaphs" as to "Mrs. Gamp" and "Mrs. Harris," that apocryphal lady being evidently confounded in hon. members' minds with Betsy Prig.

From the lay point of view, perhaps, nothing was more striking in the whole debate than the evidence clearly showing that the medical and professional aspect of the question has ceased to carry weight in the House, and that it is as a social question, involving great numbers of the poorer classes, that the registration of midwives will finally be dealt with. The undoubted interest shown by a full House in this once-shunned problem was also hopeful evidence that the time has almost come for the settlement, even if only a partial one, of a long-existing evil.

### Six Months' Private Work.

### A NURSE'S PERSONAL EXPERIENCES.

Anxious to include some experience of private nursing with my other work, I gave up a good post and attached myself to a private nursing institution in East Anglia. It was a tempting advertisement which induced me to join this particular institution, a further inducement being that it was within walking distance of my own home. I saw the advertisement on a Saturday morning, answered it, and received a reply on Monday, which was couched in very friendly, almost gushing terms, the lady principal of the institution evidently being very easy to please, or else very anxious to get a nurse, for I had sent neither references nor any particulars of training. Two days after everything was settled, and had I been free I could have gone at once. I ought to have guessed that the post had been too easily obtained to be worth having; but all unheeding, I gave up the appointment where I was happier than I had ever been, and hastened to take up this new venture.

### My Arrival.

I arrived on a Monday, and my reception by the lady principal was quite different to what her letters had led me to expect. However, I was introduced to another nurse whose room I was to share, and who proved to be nice and friendly. Our room was a small attic right up at the very top of the house, and was bare of all furniture, save our beds, a chest of drawers, and a tiny wooden washstand each. My trunks were put into another attic, which was filled with luggage, and my new friend instructed me not to unpack much but to put a few things ready in case I should be called out to a case. At four o'clock tea was served, and such a tea! I had been under the delusion that private nurses were well fed, but I was speedily undeceived. The weakest of tea, the dryest of bread, and the most indifferent butter. After this repast I fully expected I should see the lady principal again, and that she would give me some particulars as to my work, but I never set eyes on her again until I had returned from my first case. I had to get all my information from Nurse B., who was very kind, and made me feel at home as much as possible. During the evening several of the nurses dropped in, and I noticed that they one and all complained bitterly of being overworked, and said they were sick of it. So altogether I did not feel very happy when I went to bed that night. Next day I was given permission to go home, leaving, of course, my address, in case I was wanted, but I had only just got indoors and taken off my cloak and bonnet when a message came to return at once. On arrival at the Home I discovered that Nurse B. had gone to a case, and so, as I was the only nurse "in" I was not allowed to leave the premises, except the next morning, when the daughter of the superintendent gave me some errands to do for her mother.

### The First Case.

After this, I was left to my own resources, but I was not unhappy, for I had plenty of books and work. However, after two or three days I began to feel uneasy, for the maid who brought me my solitary meals on an old battered japanned tray, used to tell me strange tales of the superintendent's anger if the nurses were long out of a case. Things continued in this monotonous state until Monday afternoon, exactly a week after my arrival, when, as I was sadly speculating on things in general an interruption came in the form of the superintendent's daughter, an irritating young damsel who painted her face, wore a wig, and was the possessor of a deep masculine voice. This lady bounced into the room and told me I was to go at once to the address she gave me to help Nurse S.—one of the nurses of the institution, whom I knew slightly —with a lunatic patient. I was to hurry up and "look sharp," as a cab had been sent for. These were all the particulars vouchsafed me, and hastily putting a few things into my bag, I was ready by the time the cab came round. About half an hour's drive across a common brought us to our destination, and never shall I forget my feeling when I beheld the house. To say that my heart sank into my boots is a mild way of putting the state of my feelings, and all the wild tales of ghosts and haunted houses of which I had ever read rose to my memory. Imagine a gloomy dark-looking house, standing quite alone at the entrance to a forest, and looking as devoid of life as if it had been the abode of the princess who slept for 100 years. In blank dismay I gazed at the cabman, but of course he could not enlighten me. So after paying his fare I seized my bag, passed through the decayed-looking gate swinging on one hinge, and gave a pull at the rusty bell. There was no reply, and not a sound broke the stillness save the barking of a distant dog. I pulled again, this time with the result that the chain came out in my hands. Next I tried my knuckles, and, after a smart rapping, heard footsteps along the passage and the scraping sound of bolts being withdrawn. Then the door was opened a few inches and a woman's face peered at me, while a harsh voice demanded my business. Thinking there must have been some mistake I inquired for Nurse S., but at that moment I caught a glimpse of her in the passage, so I walked in.

### Boarders Requiring Solitude.

She informed me that the wild-looking woman who had opened the door for me was the mistress of the house who took in lady boarders requiring solitude (in truth they had it there), and the patient was one of the said boarders, who had suddenly become insane, and whose friends could not be found. Poor nurse, she was nearly worn out, so I speedily made her lie down whilst I took care of the patient. The interior of the house was quite in keeping with its outside everything was filthily dirty, thick with dust, and in the wildest state of confusion. The dirty-looking servant of the genus "slavey" appeared worked to death, and the estimable landlady seemed scared out of her wits. I had as it were to put myself in possession of the house, and find out where things were kept, as it seemed useless to depend on either the landlady or the servant for any help. The night was truly a terrible ordeal. The patient refused to be undressed or to make any preparations for retiring to rest, and the whole of the night she was walking about in her bedroom and the room adjoining, whilst I was in fear that she would make an attempt to get out of the window, or something equally unfortunate. However, the long night came to an end at last without accident, and the morning brought the doctor, and also the patient's sister-in-law from Ireland, who took everything as a matter of course, and said she had been summoned on similar errands for the last twelve years. Our next business was to convey the patient to an asylum—a troublesome and long journey, which we accomplished in a brougham. When the door of the stately mansion closed upon the poor lady I found my first private case had ended, and after returning for our bags, we were both glad to get into the Home again.

(*To be continued.*)

### Everybody's Opinion.

[Correspondence on all subjects is invited, but we cannot in any way be responsible for the opinions expressed by our correspondents. No communication can be entertained if the name and address of the correspondent are not given as a guarantee of good faith, but not necessarily for publication. All correspondents should write on one side of the paper only.]

### NURSES AND BEDSORES.

"Nurse Agnes" writes: I am very much amused at a letter written by "B. L." on this subject. I should like to ask one question, viz., What does she do with the basin of cold water? It seems to me it would be about as much use as sleeping with a cork under one's pillow to keep cramp off. I have had a good deal of experience, also success, in preventing bedsores, but I only used the *ordinary* methods of soap and warm water washing, friction, etc., taught in hospital, and known to every good nurse. This method of "B. L." sounds *extra*ordinary, and, if it is only my ignorance that makes it so, I shall be very pleased if someone will enlighten me.

[Since sending her first letter "B. L." has written again, regretting that she omitted to say, "The basin of water should be placed under the patient's bed."—Ed. The Hospital.]

### A QUESTION OF INTEREST.

"X. Y. Z." writes from Switzerland: I have had three years' training in a children's hospital and hold a first-class certificate. I now wish to get a general training, and have applied to two of the leading London hospitals for that purpose, their reply being, in both cases, that they do not take anyone to train as a probationer who has had previous training. A certificate from a children's hospital is, of course, of very little use to me, and I only began with children because I was too young for most of the best London hospitals I am now 25, and am anxious to go through the ordinary training, and wish to enter a good hospital. It seems a little unjust that because a person happens to have had a little experience in nursing children that she is therefore to be debarred from following her profession any further. I should be glad if you could tell me in your columns if it is a general rule and the reason of it.

[Although matrons frequently prefer probationers without training, as there is then nothing to unlearn, there is no general rule, and there have been very many cases in which nurses have taken general after children's training. Persevere in your applications and you will probably succeed in time. The fact of your being so far away may possibly have had an adverse influence.—Ed. Hospital.]

### "OVERWORKED MALE NURSES."

"Another Male Nurse" writes: The letter which appeared under the above title in your issue of the 22nd ult seems to me, in the first place to bear no relation whatever to its title; in fact, the writer has not said a word on the point of overwork. In the second place I think "H. W.' doet not believe in letting people do as they like, but rather that they must employ a male nurse regardless of the necessity of the case and of the circumstances of the unfortunate afflicted people. I wonder if "H. W." ever asks himself, when he is engaged on a case, the following question: How much per week must this sickness cost my employer, seeing that he pays me £2 2s.; that my board and lodging cost him about £1 per week; and that he must pay his doctor from 5s. to 10s. a visit, besides many other things necessary for the sick room, etc. This perhaps is the explanation why so many people are obliged to get a man for £20 or £30. Really, I believe "H. W." has nothing to complain of, because he says in one place that "when an employer is taken ill his friends have to send for a man who is a trained nurse", and in another place that "a year ago I nursed a gentleman through a serious illness." Why, this is simply a statement to the effect that a proper demand was met by a proper supply. What has a male nurse to do with the affairs of his late patient—what matters it to him whether he employs a man at the rate of £20 or £30 per year to wheel his bath-chair and to do odd jobs when he is not wheeling the bath-chair? And why should it concern a male nurse how the patient managed his household before he (the nurse) was called in? Or whether "attendants," "valet, draw a bath-chair, and do the duties of footman, butler, etc.?" "H.W." says that "when the employer is taken ill," they send for a "trained nurse." Accordingly the employers must be in health when their attendants "do all kinds of domestic work" for their employers, and previous to seeking his aid. I am certain that no doctor, especially "the best doctors in London and other large towns" would not let their cases, if in the least way serious, be attended to by incompetent persons—women or men. Why, the doctors' practices, their reputations and their successes depend chiefly upon the nurse! On the other hand, if "H. W." has had 17 years' experience in nursing, I should think that such an extended experience would have proved to him that people are free to choose for themselves with regard to their employees. And I would like to ask him the following question: "Why should anyone employ me at £2 2s. per week if he found that he could get a man to do all he required for £20 or £30 per annum, and his means were limited?" I am a male nurse of considerable experience and have nursed people both of the upper and middle classes. The former class have all the servants they require, and I am not suffered to do the least thing outside the pale of my duties. But as regards the latter class I very often have to do many things which are not included in the strict routine of nursing, and rather than cause any discomfort to my patient or inconvenience and extra expense to the family, I willingly do everything which is reasonable. And I am glad to say that I have not regretted this conduct in a single instance. All that I can learn from "H. W.'s" letter is this:—(1) When a gentleman is in urgent need of his services he is sent for: (2) when he has nursed his patient back to health his services are no longer required; (3) that people do not like to pay £150 per annum for services which can be purchased for £60 or £70 per annum; (4) that "H. W." is rather selfish, and that he has no sympathy with unfortunate people who are burdened with the care, anxiety, and the expense attending sickness. As to the taxation of "attendants," well, really, I see nothing in it which concerns "H. W." It is simply a question of a legal definition, and a matter which rests entirely in the hands of the Government officials.

### Novelties for Nurses.

### WHERE TO GET SHOES.

By our Shopping Correspondent.

For the benefit of my readers who are wanting new boots or shoes, I may inform them that the London Shoe Company will be selling off a large quantity of stock on Monday, March 17th, and for five following days. The sale will take place only at the City premises, 123 and 125 Queen Victoria Street, London, E.C., and not at the West End branches in Bond Street and Sloane Street, and particular emphasis is laid upon the fact that sale goods will not be sent in response to letter orders, nor will they be exchanged. This is only to be expected, for the reductions that are being made are very great. For instance, there are hand-sewn blacking leather boots, which are reduced from 25s. 9d. to 10s. 9d.; a number of glacé kid boots, which were 26s. 9d. will be 12s. 9d.; and glacé kid laced shoes, 7s. 11d. instead of 12s. 9d. I have also seen some cycling shoes, in tan, of a patent pattern, something like those usually made for men, with a buckle on the instep; these, which are light and strong, and have rubber soles, are reduced from 12s. 9d. to 4s. 11d. Tan walking shoes are 6s. 11d., and if any nurse is thinking of going to a party, I should suggest that a pair of satin shoes, at the very moderate price of 1s. (reduced from 4s. 11d.) might be just what she wants to complete her evening dress. Certainly the price cannot be quarrelled with, and as the colours are in great variety, it is probable that she may be able to find, among the 4,000 pairs, the finishing touch to her costume.

### Appointments.

[No charge is made for announcements under this head, and we are always glad to receive, and publish, appointments. But it is essential that in all cases the school of training should be given.]

Abingdon Isolation Hospital.—Miss Laurie King has been appointed matron. She was trained at the St. Helen's Hospital, where she remained for nearly four years, and has since been at the Borough Sanatorium, St. Helen's, as sister for over three and a half years.

Blackpool Infectious Diseases Hospital. — Miss Martha Gordon has been appointed night superintendent. She was trained for three years at the Western Infirmary, Glasgow, and has since been charge nurse from October, 1897, to October, 1898, at the Park Fever Hospital, London; and from October, 1898, to February, 1900, on the staff of the Edinburgh Co-operation for Trained Nurses. From February, 1900, to October, 1901, she has been at work in South Africa as a member of the Army Nursing Service Reserve.

City Hospital, Grafton Street, Liverpool.—Miss Maude C. Cumming has been appointed charge nurse. She was trained at the Hahnemann Hospital, Hope Street, Liverpool; was staff nurse for six months at the District Hospital, Yeovil; theatre nurse for twelve months at the Children's Infirmary, Myrtle Street, Liverpool; and staff nurse for six months in the men's accident and surgical ward New County Hospital, Newport, Monmouthshire.

City of Glasgow Fever Hospital.—Miss Lotty White has been appointed sister. She was trained at St. Thomas's Hospital, London, and Monsall Fever Hospital, Manchester. She has since been sister at Monsall Hospital.

Cottage Hospital, Weybridge.—Miss Laura Norman Chambers has been appointed matron. She was trained at the Metropolitan Hospital, London, for three years in connection with St. John's House, Norfolk Street, Strand, W.C., and has done nearly six years' private nursing for that institution.

Fever Hospital, Newport, Mon.—Miss Alice Rayner has been appointed sister. She was trained for three years at St. Luke's Hospital, Halifax, and has since been charge nurse at Manchester Consumption Hospital; charge nurse at Stockwell Fever Hospital and the South-Eastern Hospital, London; charge nurse at Bradford City Fever Hospital, and nurse-matron at the Cottage Hospital, Braintree.

General Infirmary, Macclesfield.—Miss Nora Elvidge has been appointed sister of children's wards. She was trained at Grimsby and District Hospital, and at Bagthorpe Hospital, Nottingham. She has since been charge nurse at Bagthorpe Hospital and night sister at Grimsby Hospital.

Hospital for Infectious Diseases, Warrington.—Miss Helen Cameron has been appointed matron. She was trained at St. Thomas's Hospital, London, and was subsequently senior staff nurse in the same institution. She has also been sister at the London Fever Hospital.

Keynsham Workhouse Infirmary.—Miss A. L. Parker Page has been appointed superintendent nurse. She was trained at Spring Hill Infirmary, Birmingham, and has since been nurse at Cheltenham and Gateshead Union Infirmaries.

Manchester Southern Hospital for Women and Children.—Miss Hannah Emily Yardley has been appointed sister. She was trained at Rotherham Hospital and Dispensary for three years and at the Royal Southern Hospital, Liverpool. She has since been staff nurse children's wards and night sister at Rotherham Hospital; nurse on the private staff of the Royal Southern Hospital, Liverpool, and on that of the Royal Berks Hospital, Reading.

Metropolitan Asylums Board, Bridge School, Witham, Essex.—Miss Marion Emerton has been appointed nurse. She was trained at Leicester Infirmary, and has since been charge nurse at the North Western Fever Hospital, the Western Fever Hospital, and Ham Green Hospital, Bristol.

Rawnboy Union Infirmary.—Miss Kate Birmingham has been appointed nurse. She was trained at Dr. Stevens' Hospital, Dublin.

Southwark Infirmary.—Miss Isabella Rippon has been appointed sister. She was trained for three years at the Royal Albert Edward Infirmary, Wigan, and for two at the Cardiff Borough Hospital. She has since been sister at the Royal Albert Edward Infirmary, Wigan, and sister at the Newport County Hospital, Monmouthshire.

Workhouse Infirmary, Great Snoring.—Miss Eleanor Layland has been appointed superintendent nurse. She was trained at the West Derby Union Infirmary, Walton, near Liverpool, and has been superintendent nurse at the Union Infirmary, Williton, Taunton.

Yorkshire Convalescent Home for Ladies, Scarborough.—Miss Edith Cobb has been appointed lady superintendent. She was trained at the London Hospital, and was subsequently superintendent of the Pendlebury Children's Hospital. She has also been district nurse at Maidenhead, Brighton, and All Saints', Surrey Square, London.

### Presentations.

Broughton Nurses Home, Manchester.—Mrs. French, of the Broughton and Kersal Nursing and Nurses' Home, Manchester, was presented by her numerous friends and admirers at the close of the "Sick Nursing Classes" with a silver tea-tray, and Crown Derby tea-set, silver chatelaine, and silver callender, in recognition of the useful and valuable instruction given to them during the course of home nursing lectures.

Cancer Hospital, Brompton.—The nursing staff and officials of the Cancer Hospital, Fulham Road, S.W., last week made presentations to two of the staff nurses who have resigned their posts on account of their approaching marriage. To Nurse Beresford, who has been at the hospital for ten years, a beautiful marble clock, suitably inscribed; and to Nurse Richards, who has been five years, they gave a handsome afternoon tea kettle. In the presentations great regret was expressed at losing companions with whom all had worked in harmony for so long, and sincere expressions of good wishes for their future happiness and prosperity were tendered.

Chelsea Infirmary.—Miss M. E. Snell, who last week resigned her post as night superintendent of the Chelsea Infirmary, has been presented with a handsome brass clock with the inscription on it: "From the nursing staff of Chelsea Infirmary," who wish her every success in her new work, which she is shortly going to commence, after a well-earned rest.

Clayton Hospital, Wakefield.—Miss Amy Eaton, who has been matron of the Clayton Hospital, Wakefield, for the last nine and a half years, was presented by the house surgeon and nursing staff with a silver mustard pot and pepperette, and by the servants with a set of silver salt cellars. Miss Eaton is leaving the Hospital to become the lady principal of the Wakefield Trained Nurses' Home.

Hamilton Branch, Q.V.J.N.I.—Miss Margaret Little, Queen's Nurse, Burnbank, having resigned her post owing to her marriage, has been presented by the committee with a handsome silver afternoon tea service. The presentation was made by the hon. president, Lady Ruthven, who referred to the great acceptance with which Miss Little had worked, and wished her happiness in her new sphere—a wish which was cordially endorsed by all the committee. The people among whom Miss Little worked also presented her with a purse of 23 sovereigns, and Dr. Watson with a very handsome gold-mounted walking-stick. This presentation was made at a social meeting in Burnbank Burgh Hall. Dr. and Mrs. Watson were later on made the recipients of very handsome presents from the "Avon" Lodge of United Oddfellows

### A Book and its Story.

### A DEAN'S DAY DREAMS.*

This charming medley of facts and fancies about the great Minster of Ely, which Dean Stubbs loves so well, is also a causerie of "the old time and the new," of parleyings as to "how heaven's high with earth's low should intertwine," which, mingled with holiday impressions of men and things, and some reflections on old legends and modern problems, form an unusually attractive book of memories. The photographs of the Minster are a valuable addition, particularly one which presents it "magic and matchless" in the moonlight, and another, giving a distant glimpse from the river, at sunset. The reader will turn instinctively also to the view "Across the garden," in which through the tracery of the trees the cathedral towers are seen. A lifelike reproduction is given of the face of a fourteenth-century predecessor of the Dean's, Prior John Crauden, from a sculptured portrait, showing a strong, sensitive, intellectual face in which power and sweetness are united in an unusual degree. The following lines, quoted in the chapter on "The Minster by Moonlight," give a vivid impression of the varying aspects in which it appears under the influence of the changing lights:—

Gold-bright in sunrise,
Gold-red in sunset,
Grey in the waning,
Kissed by the moonbeans,
Glimmering through mist cloud,
Magic and matchless.
Tower of the Lord God, Lord Everlasting.
Dreaming o'er fenland and upland and seaboard,
All through the ages guarding her heroes.

This chapter contains also a marvellously vivid impression of a moonlight musical recital held in the Cathedral. "I cannot tell you," the Dean writes, "how, sitting in that mystic, shadowed light, wrapped about with such surging waves of sound, one's very soul seemed upborne into a realm where the limits of time and space had gone—there was no more near nor far." In that strange fusion of heaven and earth solemn presences seemed to hover about us—spirits of dead men and women, "the wonderful dead who had passed through the body and gone," whose bones rest still beneath the stones of this hallowed place. "The great Queen Ætheldryth herself Virgin and Saint, first foundress of the Abbey, twelve centuries ago, Brynoth the stout, Saxon Earldoman that Homeric hero, whose death words, 'God of Nations! I thank Thee for all the joy I have had in life,' still rings down the centuries; . . . all the long line of bishops and priors, knights and warriors, chancellors and statesmen; the genius of Alan de Walsingham, monk and master-builder and flower of workmen—all lured, as it were, by the power of music, made palpable by its passion, importunate to walk the world again, seemed to our brightened senses to be filling the vast empty church with a phantom crowd of worshippers. . . . Then just as one wistfully turned . . . the prelude had ended, the moon, too, had gone . . . the magic temple, the phantom worshippers—all had vanished." A quotation from Browning's "Abt Vogler" follows, introduced to give point to reflection on the St. Ann Fugue of Bach, "in which the master-teacher strives to build for us a faith as wide as human life, deep as human need, reaching on to heaven and eternity—

On earth the broken arcs; in heaven a perfect sound . . .
. . . The high that proved too high, the heroic for earth too hard.
The passion that left the ground to lose itself in the sky,
Are the music sent up to God by the lover and the bard;
Enough that he heard it once; we shall hear it by and bye."

The concluding paragraph which follows will find an echo we believe in the hearts of many of The Hospital readers. "To-morrow will be time enough to find ourselves back again in the sober, uninteresting key of C major, and fit symbol of the common work-a-day life, the humble routine of hard duty and dull drudgery, the prosaic keynote to which, no doubt, most of our lives are set, and yet God grant the chord from which, through unpromising environment and unsatisfying effort, we may be forging these broken arcs from which one day we shall complete 'the perfect round.'"

In the cheery chapter, "The Prior's Holiday in the New World," Dean Stubbs gives his American impressions. Two years ago he took a holiday tour in the States. His notes are written in the broad genial spirit that characterises the whole book. He found the nice Americans so very nice, and the others so amusing with a kindly good humour and friendliness that was very pleasing. This cheerful good humour he attributed in a measure to the atmosphere. "How is it possible," he asks, "to be stand-offish and glum in such a brisk, bright, bracing climate? America is a land of hope, and a land of hope is of necessity one of pleasantness and good humour." His reply to a friend's query as to the title he would choose for his book on America and its people was, "I should call it 'Kith and Kin, and *More than Kind.*'" His visit was made just after the declaration of war by the Boers, and he was touched greatly by the constant expression of warm sympathy towards himself "for England's sake." "Everywhere they seemed to be saying, England stood by us in our Cuban difficulty; let us stand by her now." Among his travel treasures are a collection of menu-cards, one bearing intertwined flags had inscribed on it the following lines:—

Henceforth with mingled rays,
Our brother flags shall blaze
Through every zone.
The Union Jack shall ride,
The Stars and Stripes beside,
Proclaiming far and wide,
We two are one.

Occasionally anti-British sentiments were met with, but chiefly in newspapers of the yellow-journal type inspired by the composite elements of Irish, and German-Americanism, in centres like Chicago, where the population consists of 460,000 Germans and 245,000 Irish to 190,000 people of British origin.

Philadelphia he found a city very much to his mind. "The quaint old-fashioned substantial air of the place, its staid well-to-do respectability, its houses that were homes and not merely houses, the sedate bustle of its business streets formed an acceptable contrast to Boston and New York. Philadelphia by the people of these towns is considered a comparative city of sleep, and the story told in connection with this idea aptly illustrates their point of view. A Philadelphian girl, sprung from the old Quaker stock to which the city owes its prosperity to-day, once asked a Bostonian friend why at Boston, which is called the hub of civilisation, the streets should be so old-fashioned and crooked? 'Ah!' replied the Boston girl, with sudden illumination, 'I daresay when Boston is as dead as Philadelphia, the streets will be laid out as straight.'" Some very beautiful "In Memoriam" lines to the late President McKinley bring these varied and pleasantly-told reminiscences to a close.

---

* "In a Minster Garden." By the Dean of Ely. (Publisher: Elliott Stock, London. 1 vol. Illustrated. Price 6s. net.)

### Echoes from the Outside World.

### Movements of Royalty.

On Thursday last week the King visited the Horse Show at the Agricultural Hall, and witnessed a parade of the champions and winners, which lasted nearly an hour. The Princess of Wales had an exciting experience in the Hall on the previous day, when she presented the two gold challenge cups to the owners of the successful animals. She had barely given one to Mr. Forshaw before the winner's champion stallion, frightened by the cheers of the crowd, swung round and knocked the cup out of the prize-winner's hand. After Her Royal Highness had presented the other cup, she started with the Prince to cross from the centre of the ring towards the exit, and the crowd again cheering, the same horse became excited for the second time, and dashed in their direction. The Princess was actually in the path of the advancing animal, and but for the great presence of mind of the groom at the horse's head, who exerted all his strength to make the animal swerve on one side, she might have been terribly injured. The crowd looked on in breathless suspense, and reserved their final cheers for the Royal party until the latter had left the ring.

On Thursday the Prince of Wales attended the House of Lords and took the seat on the front cross-bench which his father used to occupy. He was present during the whole of the debate on the Scottish Public House Closing Bill, but left as soon as a division was called.

Princess Henry of Battenberg, in the capacity of Governor of the Isle of Wight, paid an official visit on Thursday to the Union and Infirmary at Parkhurst. The Princess made a tour of inspection, which was of a very thorough character, and included the imbecile, cripple, and receiving wards, the nursery—where the children sang "God Save the King"—the dining-room, and the men's and women's sitting and bed rooms. Her Royal Highness then proceeded to the Infirmary and remained for afternoon tea, which was served in the matron's room.

On Saturday the King and the Prince and Princess of Wales attended a concert given at St. James' Palace by the Abercarn Male Voice Choir, the members of which are underground miners and tinplate workers. Both the Prince and Princess wore a leek in honour of St. David's Day. After the singing of "Land of my Fathers" the Prince demanded an encore, and at the conclusion of the concert the King sent for the conductor and secretary of the choir and complimented them on the performance. Luncheon was served in the Picture Gallery in the Palace, and by the desire of the Princess of Wales each guest was presented with a box of sweetmeats before leaving.

The visit of the brother of the German Emperor to America has been marked by several interesting incidents. One of these was a luncheon given by Prince Henry of Prussia on the *Hohenzollern* to the President of the United States, and the interchange of graceful compliments between the Prince and Mr. Roosevelt. Another luncheon on a much more imposing scale, was given by the "Captains of Industry," with Mr. Pierpont Morgan at their head, to the Prince. The millionaire's lunch was preceded by the launch of the *Meteor* by Miss Roosevelt, who, on the completion of the ceremony, sent a telegram of congratulation to the Emperor. Prince Henry also sent a similar telegram, which, in the absence of a table, he wrote out upon the back of the German Ambassador, Dr. Von Holleben. Other festivities included a Press dinner on a scale of great magnificence, and a gala performance at the Opera House. This week Prince Henry, who has already escaped a collision with a runaway Washington engine, an explosion near Baltimore, and a fire at the Opera House, has been at St. Louis and Chicago, where his reception from all classes has been of a very gratifying character.

### Foreign Affairs.

The Victor Hugo Centenary, which commenced last week, has been celebrated in France with the enthusiasm which might have been expected from our neighbours on the other side of the Channel. Victor Hugo was a remarkable man of letters, and while few persons outside France will endorse the extraordinary assertion of the Mayor of Prague, that he was "the greatest poet of all time," some of his works, notably "Les Misérables" and "The Toilers of the Sea," have been widely read and appreciated in all parts of the world.

A serious accident happened to the Prime Minister of France on Friday night. When M. Waldeck-Rousseau was returning home from a Press banquet in Paris his carriage came into collision with an electric tramcar, and he was badly cut about, his hands and head being lacerated, while his shoulder was much bruised. The latest bulletins are highly favourable.

### Greater Britain.

Last week, on the anniversary of Majuba, Lord Kitchener announced the capture or slaughter of over 600 Boers—among the killed being M. Botha—with 2,000 horses, 28,000 cattle, 200 waggons, 60,000 sheep, 600 rifles, and 50,000 rounds of ammunition. The prisoners include Christian de Wet's son and his secretary, two commandants, and several field cornets. In the attack on Colonel Von Donop's convoy Lord Kitchener reports that 16 officers and 451 men of the escort were taken prisoners, of whom an officer and 105 men have since been released.

Prior to the departure of Vice-Admiral Sir Harry Rawson, the newly-appointed Governor of New South Wales, to Sydney, he was entertained last week by the Colonial Club, Sir Henry Norman in the chair. Earl Beauchamp, the last Governor of New South Wales, was present, and said he did not think that anywhere in the world finer specimens of the Anglo-Saxon race could be found than the men on the land in Australia. Sir Harry Rawson told his audience that he and his wife were going out with the intention of being heart and soul with the people.

### Parliament and Politics.

Last week the formation of a new Liberal League was announced. This is the practical outcome of the speech of Lord Rosebery at Chesterfield and Liverpool in which he intimated his abandonment of Home Rule. It has been followed by a letter from Mr. Asquith to the Chairman of the East Fife Liberal Association, in which the sometime Liberal Home Secretary, writing, apparently, as representative of a group of Liberals in the House of Commons, signifies entire concurrence in what he describes as the doctrine of the clean slate. Lord Rosebery is the first President of the new League, and Mr. Asquith, Sir Henry Fowler, and Sir Edward Grey are the first Vice-Presidents.

Notice has been given by Sir Henry Campbell-Bannerman of a motion for the appointment of a Select Committee to inquire into the War Office contracts made for the service of the Army in South Africa. This motion, which is practically a vote of censure on the Government, will be debated on Monday and Tuesday next, and will postpone the further consideration of the new Rules of Procedure. So many members of the House of Commons have suffered, or are still suffering, from influenza that the Chancellor of the Exchequer was asked on Friday night to have the Chamber disinfected. On Tuesday evening Mr. Hobhouse suggested the appointment of a Commission of experts to inquire into the nature of the disease, but the President of the Local Government Board, who has been one of its victims this year, declined to accede to the proposal.

### For Reading to the Sick.

FAITH.

"*Through Faith We Understand.*"—Heb. xi. 3.

FATHER of Lights, pure and unspeakable,
On Whom no changing shadow ever fell!
Thy light we know not, are content to see;
And shall we doubt, because we know not Thee?

*MacDonald.*

Strong Son of God! Immortal Love!
Whom we, that have not seen Thy face,
By Faith, and Faith alone, embrace,
Believing where we cannot prove. . . .
We have but Faith; we cannot know,
For knowledge is of things we see,
And yet we trust it comes from Thee,
A beam in darkness: let it grow!

*Tennyson.*

A faithful spirit does not necessarily imply a clear or deep insight into God's eternal truths, but rather the possession of a light bright enough to steer by through all the troubles and shadows of life, holding steadfastly to God's word.

A faithful spirit implies a thorough sense of dependence upon God, and a habit of seeing all things pertaining to time and to eternity in His light. Such a spirit moulds the whole life; he who possesses it will always seek first the Will of God; he will take up his cross whenever he meets it; he will mortify self-love, and close his heart to mere worldly affections, and his mind to its hollow maxims.

This spirit of faith, if acted upon throughout, will cause a rapid growth in the spiritual life. I know in Whom I have believed. You know that your love and confidence are given to your Master; do not be continually trying to manipulate yourself, in order to know how much you feel. Rest upon His Mercy. Every morning make an act disavowing intentional coldness or whatever can militate against your love for Him. After all, how everything works back to the old story—less of self, and more of God. More dwelling on His trustworthiness than on our mistrust, more energy in giving our will into His keeping. If a season comes when you appear to be unable to grasp even the very elements of the faith with any satisfying tenacity, accept the trial humbly and trustfully, wait without fretting, and it will pass in God's own time. *H. Sidney Lear.*

"The child-like faith, that asks not sight,
"Waits not for wonder or for sign,
"Believes, because it loves, aright—
"Shall see things greater, things divine.

"Heaven to that gaze shall open wide,
"And brightest angels to and fro
"On messages of love shall glide
"'Twixt God above, and Christ below."

So still the guileless man is blest,
To him all crooked paths are straight,
Him on his way to endless rest
Fresh, ever-growing strengths await.

*Keble.*

### Notes and Queries.

**The Editor is always willing to answer in this column, without any fee, all reasonable questions, as soon as possible.**

**But the following rules must be carefully observed :—**

**1. Every communication must be accompanied by the name and address of the writer.**

**2. The question must always bear upon nursing, directly or indirectly.**

**If an answer is required by letter a fee of half-a-crown must be enclosed with the note containing the inquiry, and we cannot undertake to forward letters addressed to correspondents making inquiries. It is therefore requested that our readers will not enclose either a stamp or a stamped envelope.**

*Hospital Training.*

(200) The matron of the Victoria Hospital, Folkestone, points out that there are now 50 beds. The addition to the beds is of recent date; hence the mistake.

*Minor Operation.*

(202) Can you tell a lady of limited means where she can be received for a minor operation for £1 1s., inclusive?—*Inquirer.*

Try Establishment for Gentlewomen, Harley Street, or St. Saviour's Hospital, Osnaburgh Street, N.W.

*Massage.*

(203) Will you kindly tell me what is the best place at which to get a certificate in massage.—*E. M. G.*

One of the best is given by the National Hospital for the Paralysed and Epileptic, Queen's Square, Bloomsbury, W.C. As you write from Ireland, however, the Secretary of the Society of Trained Masseuses, 12 Buckingham Street, Strand, W.C., might be able to recommend you a satisfactory teacher nearer at hand.

1. Kindly tell me if there is any place in Dublin where I could qualify in massage. 2. Also in dispensing. Which certificate ranks the higher, the English or the Irish?—*Irish.*

1. See reply to *E. M. G.* 2. Apply the Secretary of the Pharmaceutical Society, 17 Bloomsbury Square, W.C., for particulars and advice.

*Necessity of Midwifery Certificate.*

(204) I am a fully-qualified general and maternity nurse. Can I legally take confinement cases without a doctor?—*C. W.*

No. You might get yourself into sad trouble.

*Certificates.*

(205) Will you kindly tell me if a three-year certificate from a union hospital in a certain country town would qualify nurses for good posts in any Poor Law infirmary, and if there is any special form of certificate used by the Local Government Board. The probationers would be trained by a certificated nurse, and the medical officer would give them lectures and examinations?—*M. B.*

The Local Government Board would have to recognise the infirmary in question as a "training school for nurses" before it would accept the certificates as qualifying for the post of "superintendent nurse." As the institution has no resident medical officer, it is not qualified for recognition by that body.

*Heart Disease.*

(206) Will you kindly tell me how I can get a young widow with heart disease into a hospital? She has no money.—*A. E. G.*

Write to the secretary of the General Hospital, Birmingham, giving full particulars of the case.

*One Year's Training.*

(207) I am going out to South Africa next year, and should like one year's training before I go. I am not going in for the nursing profession. I am 21 years of age.—*Faith.*

You are too young for the general hospitals. You can get one year's training at the Belgrave Hospital for Children, 77 Gloucester Street, S.W. Premium £25. The Hospital for Sick Children, Great Ormond Street, would train you for three months for £1 1s. a week.

*Massage Training.*

(208) Can you tell me the names and addresses of two or three of the best hospitals in London for training massage nurses?—*M. W.*

You can be taught at the National Hospital for the Paralysed and Epileptic, Queen's Square, Bloomsbury. Or write to the Society of Trained Masseuses, 12 Buckingham Street, Strand, for advice.

**Standard Books of Reference.**

"The Nursing Profession: How and Where to Train." 2s. net; post free 2s. 4d.
"Burdett's Official Nursing Directory." 3s. net; post free, 3s. 4d.
"Burdett's Hospitals and Charities." 5s.
"The Nurses' Dictionary of Medical Terms." 2s.
"Burdett's Series of Nursing Text-Books." 1s. each.
"A Handbook for Nurses." (Illustrated). 5s.
"Nursing: Its Theory and Practice." New Edition. 3s. 6d.
"Helps in Sickness and to Health." Fifteenth Thousand. 5s.
"The Physiological Feeding of Infants." 1s.
"The Physiological Nursery Chart." 1s.; post free, 1s. 3d.
"Hospital Expenditure: The Commissariat." 2s. 6d.

All these are published by the SCIENTIFIC PRESS, Ltd., and may be obtained through any bookseller or direct from the publishers 28 and 29 Southampton Street, London, W.C.

### Travel Notes.

**By Our Travelling Correspondent.**

### XCIV.—A DISPUTED SPOT IN ROME.

### The Tarpeian Rock.

"We wandered about a long time looking for the Tarpeian Rock, less for Tarpeia's sake, than for the sake of Miriam and Donatello and the model. There are two Tarpeian rocks, between which the stranger takes his choice; and we must have chosen the wrong one, for it seemed but a shallow gulf compared to that in our fancy. We were somewhat disappointed; but then Niagara disappoints one; and as for Mont Blanc! . . ."

Thus writes W. D. Howells in his usual vein of gentle sarcasm, but it seems almost certain that the real spot is close to the Palazzo Caffarelli. It is from the courtyard of this palace that one can see it best. The exact spot described as the scene of the murder in "Transformation," existed till about 30 years ago, when the passage, door, and niche were all destroyed.

"I prefer this to any other site as having been veritably the traitor's leap," said Kenyon, "because it was so convenient to the Capitol. It was an admirable idea of those stern old fellows to fling their political criminals down from the very summit on which stood the Senate House and Jove's Temple, emblems of the institutions they sought to violate. It symbolises how sudden was the fall in those days from the utmost height of ambition to its profoundest ruin."

Then follows a description of the separation of the party, leaving Donatello and Miriam alone in the little courtyard overlooking the gulf:—

"Not so, however; not entirely alone! In the basement wall of the Palace (Caffarelli), shaded from the moon, there was a deep empty niche, that had probably once contained a statue, not empty either, for a figure now came forth from it and approached Miriam. . . . She seemed dreamily to remember falling on her knees; but in her whole recollection of that wild moment, she beheld herself as in a dim show and could not well distinguish what was done and suffered; no, not even whether she were really an actor and sufferer in that scene." . . . "The door of the little court had swung upon its hinges and partly closed itself. Hilda was quietly opening it when she was startled, mid way, by the noise of a struggle within beginning and ending all in one breathless instant. Along with it or closely succeeding it was a loud fearful cry, which quivered upward through the air and sank quivering downward to the earth. Then a silence! . . . They both leaned over the parapet and gazed downward as earnestly as if some inestimable treasure had fallen over and were yet recoverable. On the pavement below was a dark mass lying in a heap, with little or nothing human in its appearance, except that the hands were stretched out as if they might have clutched for a moment at the small square stones. . . ."

There is consummate art in the way in which the reader is made to enter into the horror of the deed, though there is no actual account of its perpetration.

### The Scala Santa.

If possible visit the Scala Santa on a Good Friday, when it presents an extraordinary appearance to one's gaze. Scores of penitents ascend and descend the 28 marble steps on their knees; no foot is allowed on them. Pope Clement XII. very wisely caused the sacred steps to be enclosed in wood, to save them, as Howells says, "from the wear and tear of devotion"! They are supposed to be the very blocks of marble which our Lord descended in leaving Pilate's house, and were brought to Rome in 326 by the Empress Helena. Close by is the Lateran Palace, for 1,000 years the home of the Popes. Very little remains to tell of its former splendour. Here in the tenth century Pope John X. witnessed the murder of his brother, and was then seized and imprisoned in the Castle of Saint Angelo by order of Marozia, who aspired to supreme power in Rome.

### The Coliseum.

It is usual to try and visit the Coliseum on a night when the moon shines brightly, but let it be in dry weather, for in times of damp the exhalations are unhealthy. The history of the Coliseum is familiar to all, and as one stands within, it is not difficult to reconstruct the scenes of savage horror, that have been enacted there. Within the last thirty years a good deal of the picturesque effect has been lost. On pretence of protecting the masonry, all the creeping plants and small bushes have been ruthlessly torn from between the stones, as if what has successfully withstood the hand of time for nearly two thousand years would be likely to collapse all at once from the presence of a little vegetable life. At the same time the large cross was removed that stood in the centre, and which commemorated the deaths of so many Christian martyrs, as well as a series of small chapels called "stations" which stood round the arena. These were used in the Via Crucis service every Friday, when a confraternity clothed in sombre garments and so masked that only their eyes were visible, like the brothers of the Misericordia, made the round of the chapels followed by a large crowd, all devoutly praying at each altar, after which a sermon was delivered by a Capuchin monk.

Madame de Stael thus speaks of the effect produced:—

"Il voulut aller au Coliseum pour entendre le Capucin qui devait y precher en plein air au pied de l'un des autels qui désignent, dans l'interieur de l'enceinte ce qu'on appelle *la route de la Croix* . . . Il est impossible de ne pas eprouver une emotion profondement religieuse."

At the time Hawthorne wrote the big cross and the chapels remained:—

"It was a strange place for song and mirth. That black cross marks one of the special blood spots of the earth, where thousands of times the dying gladiator fell, and more of human agony has been endured for the mere pastime of the multitude, than on the breadth of many battlefields. From all this crime and suffering, however, the spot has derived a more than common sanctity. An inscription promises seven years' indulgence, seven years' remission from the pains of purgatory, and earlier enjoyment of heavenly bliss, for each separate kiss imprinted on the black cross. In accordance with an ordinary custom, a pilgrim was making his progress from shrine to shrine upon his knees, and saying a penitential prayer at each. To make an end of our description, a red twinkle of light was visible amid the breadth of shadow that fell across the upper part of the Coliseum. It indicated the usual party of Americans and English paying the inevitable visit by moonlight, and exalting themselves with raptures that were Byron's, not their own."

Though now robbed of much of this picturesque charm, one must indeed have the nature of a Zoophyte not to be stirred with the memories that cluster round that sinister amphitheatre.

---

### TRAVEL NOTES AND QUERIES.

Dieppe in Winter (Sulla).—It is decidedly expensive; you would not get anything under 8s. 4d. per day at the best hotels such as you desire. The journey is very cheap, £1 17s. 3d. first return and £1 6s. 3d. second. It is easy, too, for a delicate person, there being no distance to travel after disembarking.

Perugia versus Siena (Carmen).—I fancy for a prolonged stay Siena would be preferable for several reasons. First, from what I hear, there is some slight possibility of entering a little into society there; and, secondly, there is more varied accommodation. I know of two or three native pensions, whereas at Perugia, as far as I am aware they are still non-existent. Perugia is full of charm for the archæologist and artist, and its nearness to Assisi is a great attraction, but then it has not the Cathedral of Siena. With regard to picturesque streets and architecture, there is not much to choose between them. John Addington Symonds has written some charming papers on Perugia and Siena well worth your study. Yes, Monte Olivete is within a day's excursion, full of interest, but since the death of the Abbate it has lost much of its romance. A carriage there and back will cost you about 22s.

## Figures and Tables

**Fig. 28. f1:**
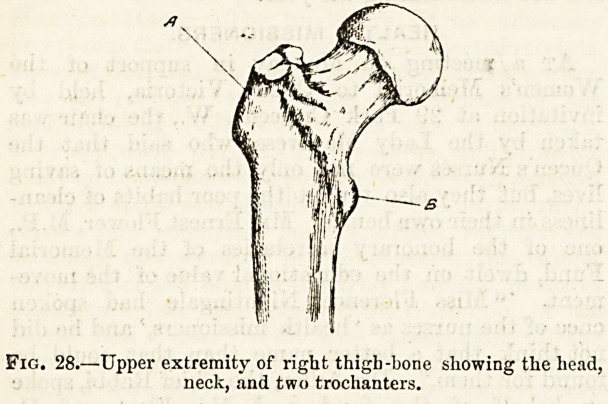


**Fig. 29. f2:**
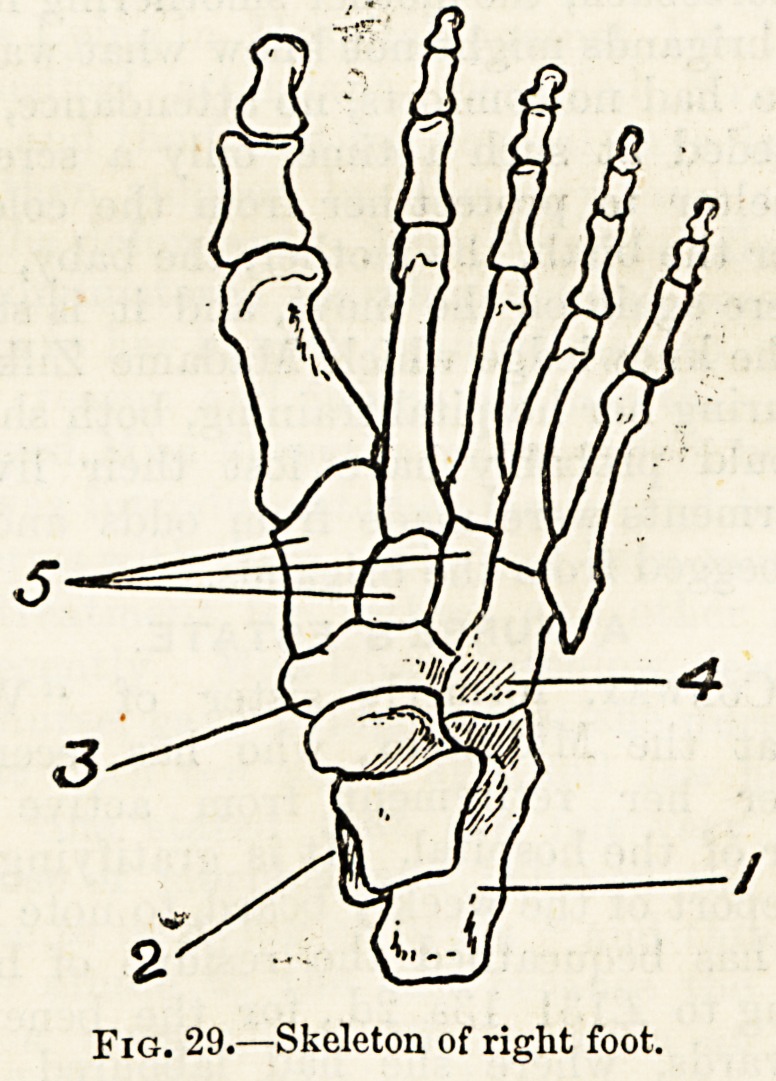


**Fig. 30. f3:**